# Coagulatory Defects in Type-1 and Type-2 Diabetes

**DOI:** 10.3390/ijms20246345

**Published:** 2019-12-16

**Authors:** Amélie I. S. Sobczak, Alan J. Stewart

**Affiliations:** Medical and Biological Sciences Building, School of Medicine, University of St Andrews, St Andrews KY16 9TF, UK; aiss@st-andrews.ac.uk

**Keywords:** atherosclerosis, endothelial dysfunction, magnesium, metal ions, microparticles, platelets, thrombosis, zinc

## Abstract

Diabetes (both type-1 and type-2) affects millions of individuals worldwide. A major cause of death for individuals with diabetes is cardiovascular diseases, in part since both types of diabetes lead to physiological changes that affect haemostasis. Those changes include altered concentrations of coagulatory proteins, hyper-activation of platelets, changes in metal ion homeostasis, alterations in lipid metabolism (leading to lipotoxicity in the heart and atherosclerosis), the presence of pro-coagulatory microparticles and endothelial dysfunction. In this review, we explore the different mechanisms by which diabetes leads to an increased risk of developing coagulatory disorders and how this differs between type-1 and type-2 diabetes.

## 1. Introduction

Diabetes is a term used to describe a group of conditions that impact upon the body’s ability to properly control blood glucose levels. In 2017, combined occurrences of type-1 diabetes mellitus (T1DM) and type-2 diabetes mellitus (T2DM) were estimated at 425 million individuals worldwide [1]. This number is predicted to rise to 629 million by 2045 [1]. All forms of diabetes are characterised by defective signalling of insulin, the peptide hormone responsible for stimulating cellular glucose uptake. In T1DM, the insulin-secreting β-cells in the islets of the pancreas are destroyed, by the immune system in the first subtype of T1DM, idiopathically in the second subtype [2]. This results in a decrease of insulin production. T2DM is a polygenetic disease; it can be divided into two subtypes, with and without obesity, and several genes can predispose individuals to developing the disease. It displays a heterogenous phenotype that is the consequence of resistance to insulin signalling, often due to defects associated with insulin receptors. At the beginning of the disease, insulin secretion is impaired and results in hyperinsulinemia. However, as the disease progresses, β-cells can become damaged, leading to hypoinsulinemia [3].

Both T1DM and T2DM have wide-ranging consequences for the body as glucose levels are associated with many physiological processes. These include lipid metabolism and the regulation of inflammation, vasodilatation, basic cell growth and replication. Unmanaged diabetes and hyperglycaemia can worsen these physiological changes, potentially leading to diabetes-associated complications. In particular, individuals with diabetes are two to three times more likely to develop cardiovascular diseases than those without diabetes [1]. For example, several coagulatory defects are observed in individuals with T1DM or T2DM. Indeed, the vascular endothelium is altered in individuals with both these types of diabetes, and so hypertension, premature atherosclerosis and more extensive vascular diseases can be found in affected individuals compared to the general population, thus also increasing their risk of plaque rupture (in the case of atherosclerosis) and thrombus formation [4,5,6]. Furthermore, in individuals with diabetes, platelets are hyper-reactive, giving rise to increased activation of prothrombotic factors and decreased fibrinolysis which results in an increased risk of thrombosis [4,7]. In addition, the altered lipid profile found in individuals with diabetes affects cardiac function and can cause lipotoxicity in the heart [5]. Due to these factors, up to 80% of individuals with diabetes die as a result of cardiovascular complications [8]. The prognosis following a cardiovascular event remains poor for individuals with diabetes despite intensive research on the subject and the development of new therapies [4,9]. Thus, it is important to better understand the underlying mechanisms that drive the haemostatic changes observed in T1DM and T2DM.

Despite the known increased risk of cardiovascular disease in individuals with diabetes, the pathophysiology underlying this relationship is complex and not completely understood. Nevertheless, among the many physiological changes induced by diabetes that can impact on the cardiovascular system are changes in the concentrations of plasma proteins and metal ions, altered lipid metabolism and lipid composition (resulting in altered metabolic regulation), cardiac lipotoxicity and atherosclerosis, endothelial dysfunction, platelet hyper-activation and the presence of pro-coagulatory particles in the blood. Here, we review the molecular and cellular changes that can lead to the increased thrombotic risk observed in individuals with diabetes.

## 2. Diabetes and Thrombosis: Abnormal Coagulation Mechanisms

### 2.1. Alterations of Plasma Protein Concentrations

**Figure 1:**
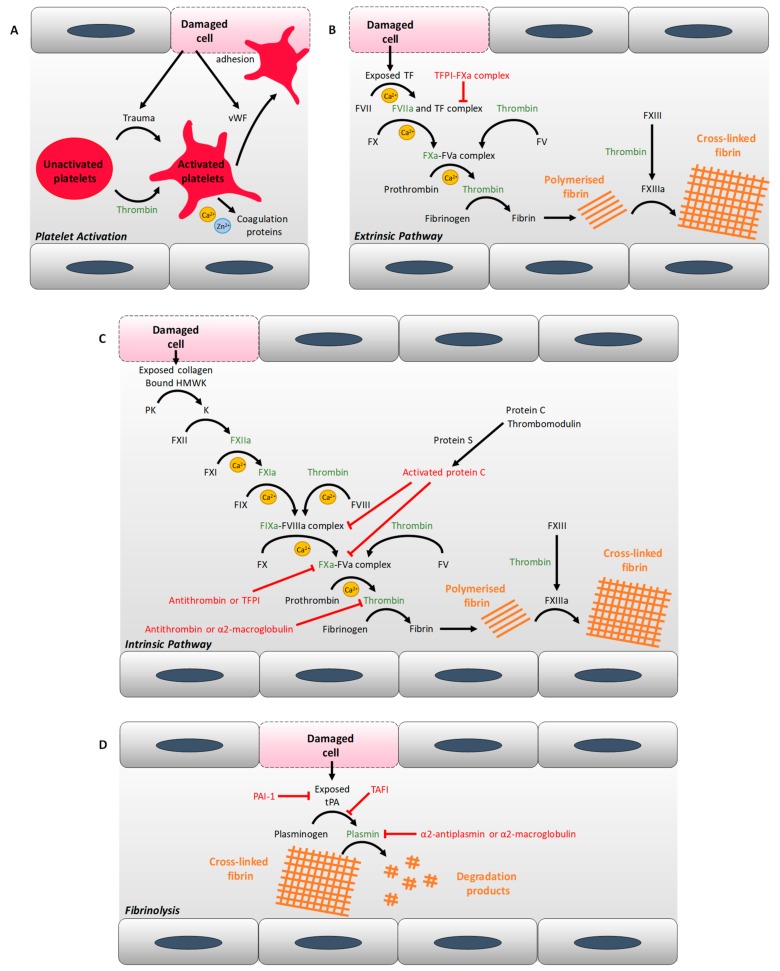
Simplified schema of coagulation–activation, anticoagulation, fibrinolysis and anti-fibrinolysis by various mechanisms. (**A**) Platelet activation. (**B**) Extrinsic pathway of coagulation. (**C**) Intrinsic pathway of coagulation. (**D**) Fibrinolysis. Anticoagulation and anti-fibrinolytic activities are indicated in red. All proteins inhibited by antithrombin are indicated in green. Abbreviations used: F, coagulation factor; HMWK, high molecular weight kininogen; K, kallikrein; PAI-1, plasminogen activator inhibitor-1; PK, pre-kallikrein; TAFI, thrombin-activatable fibrinolysis inhibitor; TF, tissue factor; TRPI, tissue factor pathway inhibitor; tPA, tissue plasminogen activator; vWF, von Willebrand factor. Activated coagulation factors are indicated with an “a”.

T1DM and T2DM are associated with changes in blood coagulability, including alterations in clot structure and in the kinetics of clot formation and lysis. The factors responsible for these alterations include changes in the concentration and activity of numerous coagulatory proteins, resulting in defective thrombin generation and changes in the molecular make-up of fibrin clots. Proteins identified as exhibiting an altered concentration in both T1DM and T2DM are summarised in Table 1, while Figure 1 summarises the activity of those proteins in coagulation. Proteins with elevated concentrations in both types of diabetes include von Willebrand factor (vWF) [10,11,12], (pre)kallikrein [13,14], factor V [15], (activated) factor VII [15,16,17], factor VIII [15,18], factor X [15], factor XI [14], prothrombin [15], and fibrinogen [19,20,21] (although a study has also reported its reduction in T1DM [22]). Proteins only elevated in T2DM include: kininogen [23], soluble tissue factor [16,24], factor IX [18], (activated) factor XII [18,25], and factor XIII [26]. In contrast, in T1DM, activated factor XII levels are reduced [27]. Simultaneously to changes in pro-coagulation proteins, several anticoagulation proteins have a reduced plasma concentration in both types of diabetes, including protein C [15,19,28,29] and protein S [30], but thrombomodulin [29,31] has an elevated concentration in both types of diabetes and tissue factor pathway inhibitor levels are elevated in T2DM [16]. A number of reports have examined antithrombin concentration in T2DM. One such study found reduced concentrations [32], whilst two other studies reported elevated concentrations of this protein associated with the disease [18,33]. The cause of this difference is not known; it may be due to a difference in methodology or to the individuals studied being at a different stage of progression of the disease. Antithrombin cofactors, the heparan sulphate glycosaminoglycans in the endothelium surface layer, are largely responsible for the anticoagulant properties of the endothelium [34]; the concentration of these molecules is decreased in the arteries of individuals with T2DM, especially in those with lesions [35]. The pro-fibrinolysis protein, tissue plasminogen activator has an increased concentration in individuals with glucose intolerance [36] and in individuals with T1DM [37] or T2DM [24], but its availability is decreased because of the elevated concentration of plasminogen activator inhibitor 1 (PAI-1) associated with glucose intolerance in non-diabetic individuals [36] and with individuals with T2DM [10,19,20,24,38]. In contrast, in T1DM, the PAI-1 concentration is reduced [39]. The concentration of other inhibitors of fibrinolysis, including thrombin-activatable fibrinolysis inhibitor [40,41] and α2-macroglobulin [32,42], are also elevated in both T1DM and T2DM, while the α2-antiplasmin concentration is elevated in T2DM [19], but in T1DM it has been reported to be elevated in two studies [19,43], but reduced in another [22]. Again, the origin of this difference is not known but could be due to differences in methodology or to different stages of progression of the disease in the individuals studied. Changes in plasma protein concentrations as well as plasma glucose levels lead to an increase in plasma viscosity in T2DM [44] and an increasing trend has been measured in T1DM, especially in individuals with bad glycaemic control [45,46]. Most but not all changes in the protein concentration of coagulatory proteins in plasma are due to uncontrolled glycaemia and so can often be reversed through control of blood glucose levels: for example, protein C, protein S and antithrombin concentrations have been demonstrated to increase in T2DM subjects following improvement in glycaemic control [47]. Some proteomic alterations are influenced by genetic factors. For example, the increase in fibrinogen concentration and factor VII coagulant activity in T2DM are also seen in first degree relatives of individuals without the disease [48]. In addition, chronic inflammation (as is associated with both T1DM and T2DM) leads to activation of both the complement system and the kinin–kallikrein system, resulting in the activation of factor XII and elevated concentrations of several proteins including factor VIII, tissue factor, prothrombin and fibrinogen [7].

The concentration of coagulatory proteins is not the only factor that impacts on coagulation. While elevated concentrations of PAI-1 are found in T2DM [10,19,20,24,38], in T1DM it is the activity of the protein that is reduced [22,52]. Also, alterations in fibrin(ogen) function are also more complex than just a change in concentration: an examination of clots formed from fibrinogen purified from individuals with T2DM and controls found the T2DM-derived samples exhibited denser and less porous clots [53]. This can be explained by elevated glycation of fibrinogen in diabetes and can be abrogated with better glycaemic control [53,54,55]. Fibrinogen is not the only protein affected by poor glycaemic control; plasminogen, the precursor of plasmin, undergoes increased glycation in individuals with T1DM, thereby leading to a reduced fibrinolytic-activity of plasmin [51]. Furthermore, the activity of antithrombin is inhibited by methylglyoxal, a by-product of hyperglycaemia [56]. In addition, in healthy individuals subjected to combined hyperglycaemia and hyperinsulinemia, the tissue factor pathway has been shown to be increasingly activated (compared to an euglycemia–hyperinsulinemia group), as reflected by elevated concentrations of activated factor VII and tissue factor pathway inhibitor, as well as in an increase in factor VII activity [57]. Thus, thrombin generation (followed by measuring levels of the thrombin–antithrombin complex) is increased in individuals with T2DM or T1DM [28,58]. Good glycaemic control is also important for anticoagulant activity, with better glycaemic control in T2DM leading to a reduction of thrombin generation [59] and an increase of the anticoagulant activity of antithrombin, protein C and protein S [47].

Thus, changes in both the concentrations and activities of coagulation proteins have important consequences on fibrin clot formation, clot lysis parameters and fibrin clot ultrastructure. Elevated PAI-1 concentration results in prolonged lysis time of the fibrin clot in individuals with T2DM [10]. The elevated concentration of complement protein C3 found in T1DM results in the protein being increasingly incorporated into fibrin clots formed from fibrinogen purified from blood from those individuals with T1DM, leading to delayed fibrin clot lysis [60]. This has also been observed in T2DM [61]. In T1DM, both lysis time and the concentration of C3 improved with better glycaemic control [60]. Similarly, α2-antiplasmin is also increasingly incorporated into fibrin clots in T1DM and T2DM, which has been shown to increase lysis resistance [22,62]. Fibrin clots formed in individuals with T1DM, like with T2DM, are more compact in correlation with glycaemic control [63]. In both types of diabetes, fibrin clots are more resistant to fibrinolysis [10,53,63]. Diabetes duration also has an impact, with prolonged T2DM duration (more than five years) associated with increased thrombin generation, reduced fibrinolysis and a pro-thrombotic phenotype even with good glycaemic control [64]. PAI-1 and t-PA antigen levels are also higher in prolonged T2DM duration while fibrinogen, plasminogen, soluble thrombomodulin and thrombin-activatable fibrinolysis inhibitor antigen levels are unaffected [64]. In addition, differences in coagulatory protein levels and clot parameters were found between males and females with T1DM or T2DM: indeed, fibrinogen and PAI-1 concentrations are higher in females with T2DM than in males with T2DM, and after correcting for those factors, females still had more compact clots that were resistant to fibrinolysis than males [65], However, another study with fewer individuals found unchanged fibrinogen levels and reduced PAI-1 levels in females with T2DM [66]. The incorporation of α2-antiplasmin in clots was found to be increased in females with T2DM, making them more resistant to fibrinolysis [66]. In T1DM, clot density and fibrinogen concentration in females are the same as in males, while their factor XIII concentration is higher than in males [67]. PAI-1 concentration is also similar between females and males with T1DM [39]. When looking at a younger cohort with T1DM (under 30 years old), females have a prolonged lysis time compared to males, but this is not the case for an older cohort [67]. Thus, in both types of diabetes, these combined changes result in an increase in pro-coagulation mechanism and a decrease in anticoagulation and fibrinolysis, leading to an elevated thrombosis risk.

### 2.2. Changes in Metal Ion Homeostasis

Metal ions play numerous roles in blood plasma, which include structural and catalytic functions. The plasma concentration of several metal ions is known to be altered in T1DM and T2DM [68]. This is important as many of these are necessary for the normal functioning of proteins involved in coagulation [69,70,71,72,73]. Ca^2+^ is an important regulator of coagulation. Ca^2+^ is released by activated platelets and is required for clotting to take place (in particular for tenase and prothrombinase complexes to function). Chelating agents that bind calcium (e.g., citrate and ethylenediaminetetraacetic acid) are common anticoagulants used when taking blood samples. Several studies have found associations between high levels of calcium in the blood and risk of developing T2DM. Such studies include the PREDIMED study [74], the Atherosclerosis Risk in Communities (ARIC) study [75], the Insulin Resistance Atherosclerosis study [76] and the Tromsø study [77]. In addition, elevated total plasma calcium levels are also found in individuals with T2DM compared to healthy controls; no difference was observed between males and females and the duration of diabetes had no influence on calcium levels [78]. Serum calcium levels are unchanged in T1DM [79,80]. The effects of elevated calcium levels on coagulation in T2DM have not been fully characterised. In the general population, a meta-analysis has shown that taking high levels of calcium supplements (>1000 mg/day) increases cardiovascular risk in men but not in women where the benefits of taking the supplements outweighs the risks [81]. In this study, calcium supplementation was found to also increase the risk of coronary artery calcification and atherosclerosis in both sexes [81]. However not all calcium taken in supplements will be absorbed and much of the absorbed excess calcium will be stored in bones. In individuals with T2DM, plasma calcium levels are elevated. Thus, further studies are necessary to determine if part of the hypercoagulability found in individuals with T2DM may be explained by alterations in plasma calcium concentration.

In addition to Ca^2+^, Zn^2+^ is also extremely important in regulating coagulation [69]. Like with Ca^2+^, Zn^2+^ is also released by activated platelets, as well as damaged epithelial cells and atherosclerotic plaques; it is also contained by neutrophils, lymphocytes and erythrocytes and may therefore be released at sites of injury (although this has yet to be confirmed) [82]. Zn^2+^ is involved in all steps of coagulation: pro-coagulatory, anti-coagulatory, pro-fibrinolysis and anti-fibrinolysis mechanisms, as well as platelet activation and aggregation [69]. Zinc deficiency causes bleeding and platelet aggregation disorders [69]. In T2DM and T1DM, zinc concentrations are reduced compared to healthy subjects [83,84]. However, in plasma, Zn^2+^ ions are transported by serum albumin, which is also the main transporter of free fatty acids (FFAs) [85]. When a FFA binds to the high affinity binding site FA2 on serum albumin, the protein conformation changes and the main Zn^2+^ binding site is disrupted and can no longer bind Zn^2+^ [85]. Thus, when pathological concentrations of FFAs are present in the blood, such as is the case in T2DM and in some cases of T1DM, Zn^2+^ handling/buffering by serum albumin is dysregulated and plasma zinc speciation (the molecules to which it is bound) is altered [86]. Recently we have shown using size-exclusion-chromatography-ICP-MS that Zn^2+^ is redistributed among other plasma proteins in the presence of pathophysiological FFA concentrations [87]. Considering the many coagulatory proteins that are regulated by Zn^2+^, this altered zinc speciation can dysregulate coagulation, resulting in increased platelet aggregation, increased fibrin clot density, and delayed fibrinolysis, thus potentially participating in the elevated thrombotic risks found in T2DM [86].

Mg^2+^ is also important for coagulation. Mg^2+^ can potentiate the activation of factor X by activated factor IX while in the presence of activated factor VIII, phospholipids and Ca^2+^ [88], the activation of factor IX by activated factor VII-tissue factor complex [88], and the inactivation of activated factor V by activated protein C [89]. Mg^2+^ also affects clot time by accelerating clotting at low concentrations and slowing or completely preventing fibrin clotting at high concentration (as it competes with Ca^2+^ for binding to coagulation factors [90,91,92,93]). Furthermore, Mg^2+^ shortens fibrin clot lysis time, possibly through an inhibition of PAI-1 in the presence of thrombin and vitronectin [92]. Magnesium deficiency in humans and animals has been shown to cause hypercoagulability [94]. Magnesium deficiency has been observed in T1DM in both males and females, although the effect is more pronounced in females [68]. Magnesium deficiency in T1DM has been associated with delayed fibrinolysis and a higher thrombotic risk [39]. Individuals with T2DM are also at risk of magnesium deficiency and may therefore be affected by a similar mechanism [95]. Bad glycaemic control is associated with magnesium deficiency, as it reduces the tubular reabsorption of magnesium [68].

Total plasma copper levels are elevated in T2DM and T1DM [83,96]. The potential effect of copper on coagulation is not well known, despite Cu^+^ and Cu^2+^ being essential cofactors of several coagulation proteins (e.g., coagulation factors V and VIII) [72,73]. Elevated dietary levels of copper in rats (which were reflected in copper concentrations in the liver) were found not to affect clot time when clotting was induced by thromboplastin and Ca^2+^ (when assaying for factor X, thrombin and fibrinogen activation) or Ca^2+^ and phospholipids (when testing the whole extrinsic pathway of coagulation) [97]. However, more than 95% of copper found in plasma is carried by the protein ceruloplasmin [71]. Levels of ceruloplasmin are elevated in individuals with T1DM, possibly due to inflammatory processes [96]. In T2DM, ceruloplasmin levels can be reduced or elevated; a meta-analysis has shown that globally ceruloplasmin levels are increased but not significantly (*p* = 0.06) and that sex does not influence this parameter [83]. However, another study compared T2DM individuals without diabetic complications with T2DM individuals with complications and found the latter group to have higher ceruloplasmin levels [98]. In cases where high blood concentrations of ceruloplasmin has been observed, the protein has been shown to bind to activated protein C to reduce its anticoagulant activity and induce acquired activated protein C resistance—a state associated with a higher risk of venous thrombosis [70,71]. No direct study on the link between ceruloplasmin levels in diabetes and coagulation has been performed.

Individuals with T2DM have higher plasma levels of ferritin and higher, but not significantly so, total plasma levels of iron (*p* = 0.06); sex does not influence either parameter [83]. Individuals with T1DM are deficient in iron; diabetes duration or sex had no influence on iron deficiency, but the menstrual cycle did [99,100]. For those individuals deficient in iron, treatment includes supplementation with Fe^3+^ salt [101]. This has been shown to impact on coagulation by extending the clotting time of plasma (possibly by competing with calcium in binding coagulation factors), weakening the fibrin clot (by interacting with fibrinogen and fibrin), and inducing the precipitation of plasma proteins to form “insoluble coagulums” resistant to lysis (notably by binding and degrading serum albumin and possibly transferrin), thus increasing the risk of thrombosis [101]. In addition, Fe^3+^ has been shown to initiate the conversion of fibrinogen into a fibrin-like polymer, parafibrin, that is resistant to proteolysis and so is deposited in blood vessels [102]. The persistent presence of this parafibrin has been argued to cause chronic inflammation [102]. Thus, the altered levels of plasma metal ions in individuals with T1DM and T2DM will impact on coagulation and the risk of developing cardiovascular diseases.

### 2.3. Changes in Lipid Metabolism at the Origin of Atherosclerosis and Lipotoxicity

Both T1DM and T2DM are associated with changes in lipid metabolism. Plasma cholesterol, low-density lipoprotein (LDL) and triglyceride concentrations are increased and high-density lipoprotein (HDL) concentration is decreased in individuals with T2DM, and in individuals with T1DM and bad glycaemic control [103,104]. Unchanged cholesterol levels in individuals with T1DM and good glycaemic control can be deceptive as lipid profiles and functioning are altered [105,106]. Traditionally, high levels of total cholesterol and LDL have been regarded as a major risk factor of atherosclerosis and cardiovascular disease in the general population. However, a recent review of the literature by Ravnskov et al. has argued that total cholesterol and LDL do not cause those diseases [107]. They explain the difference between this new view and the traditional view as the failure of most meta-analyses to properly account for negative studies [107]. They also argue that the associations between cardiovascular disease and LDL or cholesterol concentrations found in certain cohorts can be explained through different mechanisms. A possible explanation is that infections can cause cardiovascular disease and that LDL participates in immune functioning by adhering to and inactivating microorganisms and their toxic products [107]. Another is that stress also causes cardiovascular diseases as increased production of adrenalin and noradrenaline contribute to hypertension and hyper-coagulation, and that cholesterol is a precursor for cortisol and other steroid stress hormones [107]. However, whether this new view reflects what really happens at the molecular level is unclear as this study looked at the general population and not individuals with T1DM and T2DM, in which lipid-lowering drugs remain an essential treatment to prevent the development of complications, including cardiovascular diseases [104,105].

In diabetes, high LDL levels are associated with cardiovascular diseases, but they are not an accurate predictor of cardiovascular risks in T1DM [105,108,109]. Nevertheless, there are higher levels of “small dense LDL” in T1DM and T2DM and these forms of LDL penetrate more easily in the arterial wall than “large buoyant LDL” [110,111,112]. Small dense LDL are also more susceptible to oxidative stress, have a reduced affinity for LDL receptors and have a prolonged half-life in plasma than large buoyant LDL [110]. In addition, they are more easily glycated as they carry a higher proportion of apolipoprotein B, which is exposed to glucose [113]. Furthermore, oxidized LDL inhibits endothelial nitric oxide production [114] and can more easily be taken up by macrophages as part of atherosclerotic plaque formation [112]. These characteristics are all associated with endothelial dysfunction (see Section 2.4) and cardiovascular diseases [105,112,115].

HDL has long been thought to have protective properties against cardiovascular diseases. However, recent evidence has shown that a low HDL concentration is associated with an increased cardiovascular risk, but that a high HDL concentration does not have a protective effect and could even be dangerous, as has been shown in the general population and in individuals with T1DM [106,116,117]. These unexpected results may be explained by the existence of different HDL subspecies with different functions [118]. Thus, it may be beneficial to directly measure HDL function, such as its role in promoting reverse cholesterol transport (the net movement of cholesterol from peripheral tissues to the liver to be excreted through the bile) [118]. For example, when the macrophages present in artery walls accumulate excess cholesterol, the ATP-binding cassette transporters A1 and G1 are induced and this results in the efflux of cholesterol from the macrophages to the HDL [118]. HDL efflux has been found to be a better predictor of cardiovascular disease than HDL levels [119,120,121,122], although other studies have disputed this [123,124,125,126]. HDL efflux is reduced in individuals with T2DM [127,128] or T1DM [129]. This may be because reactive oxygen species, which are increased in individuals with diabetes, can impact HDL function [130]. Beyond its role in cholesterol efflux, HDL also has an anti-atherosclerotic effect as it promotes nitric oxide production in endothelial cells, an essential feature for endothelial function [118]. HDL also has anti-inflammatory and antioxidant effects [118]. A study comparing the effects of HDL taken from individuals with T2DM and controls showed that in T2DM, HDL failed to stimulate nitric oxide production by endothelial cells and did not promote endothelial repair [131]. In addition, in T2DM, HDL have reduced levels of HDL-associated sphingosine-1-phosphate (S1P), resulting in a reduced ability to activate endothelial nitric oxide synthase [132]. Furthermore, individuals with T1DM have the same levels of S1P and apolipoprotein M in total HDL as controls, but the HDL-associated apolipoprotein M/S1P complex move to a different subset of HDL, from buoyant HDL to dense HDL, where it has reduced anti-inflammatory effects due to altered S1P_1_ receptor activation [133].

Hypertriglyceridemia is often associated with T1DM and T2DM [134,135,136]. Cholesterol ester transfer protein (CETP) exchanges cholesterol and triglycerides between very low-density lipoprotein and HDL [137]. When the plasma level of triglycerides is too high, the equilibrium is displaced, and HDL are impoverished in cholesterol and enriched in triglycerides [137]. This results in impaired HDL structure, as the hydrophobic core of the triglycerides partially extrude to the HDL surface [138], and therefore impaired HDL function [139], and (eventually) endothelial dysfunction. Contrary to HDL-cholesterol levels, HDL-triglycerides have been shown to be an effective marker of increased cardiovascular risk in the general population [140] and in individuals with T2DM or with metabolic syndrome [137].

**Figure 2:**
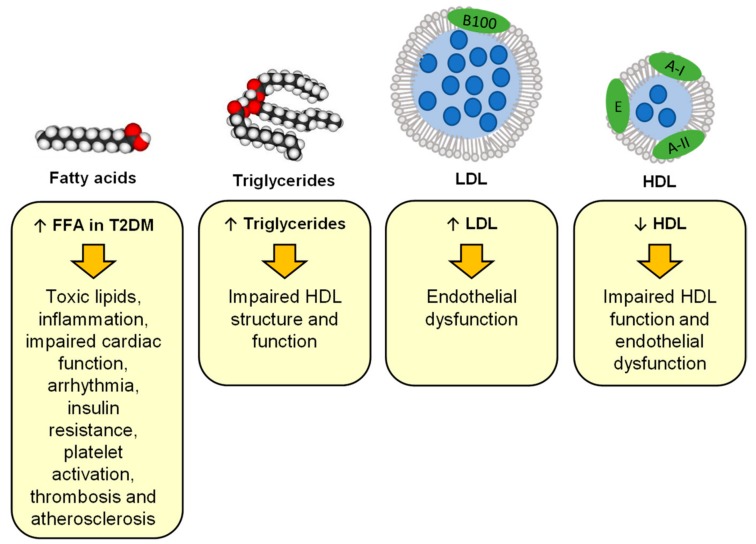
An altered lipid profile increases the risk of cardiovascular disease in T1DM and T2DM. Abbreviations used: FFA, free fatty acid; HDL, high-density lipoprotein; LDL, low-density lipoprotein; T2DM, type-2 diabetes mellitus.

FFA levels are also altered in diabetes; total plasma FFA concentrations and the plasma concentrations of most major FFA species are increased in individuals with T2DM [86], while they are reduced in individuals with T1DM and bad glycaemic control [141]. FFAs are important regulators of many physiological processes and their dysregulation can have important consequences [5]. The most obvious one is the adherence of excess FFAs on the endothelial walls of blood vessels and their subsequent accumulation that results in the formation of atherosclerosis plaques [5]. These plaques can make blood vessels narrower, thus facilitating their full blockage, and plaque rupture is a pro-thrombotic event that can trigger thrombosis and embolism [142]. In addition, the fatty acid translocase CD36, which is located on macrophages and platelets, can be activated by FFA to trigger coagulation [143]. Furthermore, elevated plasma FFA concentrations directly affect fibrin clot parameters; the saturated FFA, stearic acid, has been shown to increase the diameter of fibrin fibres in a purified system, while an unsaturated FFA, oleic acid, reduced it [144]. Stearic and oleic acids also both increased clotting time and reduced the mechanical stability of the clot through a decreased rigidity, a higher deformability and a decreased internal resistance to shear stress [144]. In addition, plasma FFA levels also impact on the function of the heart as they increase its susceptibility to oxidative stress and ischemic damage [145]. Indeed, excess FFAs also lead to the formation of toxic lipids (in particular diacylglycerides and ceramide) thus promoting endoplasmic reticulum stress, mitochondrial dysfunction and the generation of reactive oxygen species [5]. This leads to inflammation, insulin resistance and apoptosis of cells [5]. These toxic lipids also activate protein kinase C (PKC) [5]. PKC activation impairs intracellular Ca^2+^ handling in the heart, affecting cardiac contractibility and promoting cardiac fibrosis and hypertrophy [145,146]. While n-3 unsaturated FFAs have been shown to have anti-arrhythmic and cardioprotective effects, saturated FFAs can lead to electrophysiological remodelling and sustained and fatal arrhythmias [147]. Thus, the changes in lipid levels and lipid metabolism found in T1DM and T2DM can have a strong impact on the risk of developing cardiovascular diseases, as summarised in Figure 2.

### 2.4. Endothelial Dysfunction

**Figure 3:**
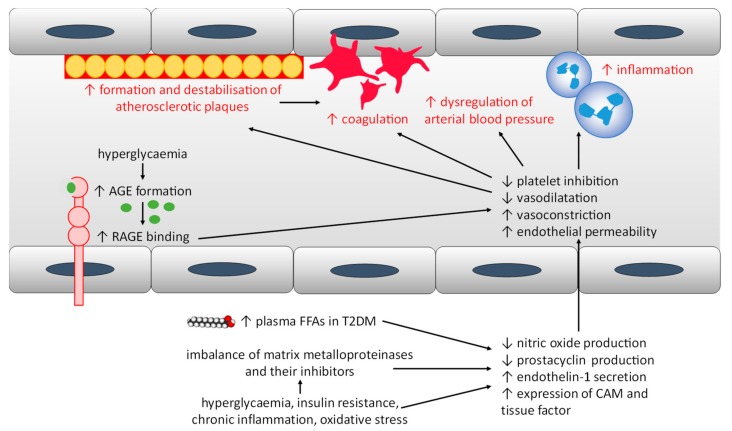
The changes in inflammation, oxidative stress and blood levels of glucose, insulin and lipids associated with T1DM and T2DM cause endothelial dysfunction, which itself results in an increase in the risk of cardiovascular disease. Abbreviations used: AGE, advanced glycation end-products; CAM, cell adhesion molecules; FFA, free fatty acid; T2DM, type-2 diabetes mellitus; RAGE, advanced glycation end-product receptors, T1DM, type-1 diabetes mellitus; T2DM, type-2 diabetes mellitus.

Endothelial dysfunction can be defined as a diminished production and/or availability of nitric oxide, a molecule involved in vascular homeostasis, vasodilation and platelet inhibition, and as an imbalance between vasodilators and vasoconstrictors in the vasculature. Endothelial dysfunction precedes the development of atherosclerosis and increases the risk of cardiovascular diseases. Diabetes is associated with a series of changes in endothelial function caused by several factors including an excess of plasma FFAs in T2DM and alterations in glucose metabolism, impaired insulin signalling, chronic inflammation and oxidative stress in both T1DM and T2DM [148]. Excess plasma FFAs causes a dysregulation of Ca^2+^ and insulin signalling, resulting in a reduction in the production of nitric oxide, thus leading to increased endothelial permeability [142]. The activation of the NLRP3 inflammasome by excess FFAs also contributes to this increase in permeability [142]. Excess FFAs also impact on the renin–angiotensin system, resulting in the dysregulation of arterial blood pressure [142]. In addition, the activation of the NF-κB inflammation pathway (by saturated FFAs but not polyunsaturated FFAs) leads to an increase in the production of superoxide in the endothelium [142], which itself increases the concentration of a range of enzymes including oxidative enzyme systems such as NADPH oxidase, xanthine oxidase, cyclooxygenases, lipoxygenases, myeloperoxidases, cytochrome P450 monooxygenase, uncoupled nitric oxide synthase, and peroxidases. Collectively, these enzymes inactivate nitric oxide. Furthermore, the combination of oxidative stress and hyperglycaemia seen in diabetes leads to the glycation of plasma proteins and lipids and generates advanced glycation end-products (AGEs) [148]. These AGEs then accumulate in the vessel walls and disrupt cell function, notably by binding to AGE receptors (RAGEs) [148]. Signalling by RAGEs downregulates nitric oxide synthase in endothelial cells and upregulates expression of vascular cell adhesion molecule, intercellular adhesion molecule, E-selectin (three cellular adhesion molecules), monocyte chemoattractant protein-1 (a regulator of migration and infiltration of monocytes and macrophages), endothelin-1 (a vasoconstrictor) and tissue factor [148]. Insulin resistance itself also reduces nitric oxide production and stimulates endothelin-1 secretion [149]. Dysregulation of the aforementioned pathways contributes to the pro-inflammatory and pro-thrombotic properties of the endothelium in diabetes. In addition, diabetes is associated with decreased endothelial synthesis of prostacyclin, a vasodilator and inhibitor of platelet activation [148]. Furthermore, matrix metalloproteinases are zinc-binding proteinases that degrade components of the extracellular matrix and whose production is upregulated notably by hyperglycaemia, pro-inflammatory mediators and reactive oxygen species [150]. Matrix metalloproteinase levels are associated with cardiovascular disease development and all-cause mortality in T1DM [151,152,153], and with cardiovascular organ damage in T2DM [154]. In diabetes, they increase inflammation, endothelial dysfunction, vascular remodelling and thrombus formation [155,156]. The imbalance of matrix metalloproteinases and their inhibitors observed in diabetes is associated with the formation and destabilisation of atherosclerotic plaques [157,158]. Thus, endothelial dysfunction is a major factor contributing to the risk of cardiovascular disease in individuals with T1DM and T2DM, as summarised in Figure 3.

### 2.5. Platelet Hyper-Activation

Many of the key changes that impact upon the coagulation system in diabetes involve platelets. In normal physiology, platelets are activated in response to exogenous stimuli including thrombin (which binds to the G protein-coupled receptors, PAR1, PAR3 and PAR4), collagen (which binds to the receptor GPVI-αIIβI) and thromboxane A2. The P2Y_12_ pathway can amplify these stimuli by triggering the secretion of thromboxane A2 and ADP from internal stores. Activation is carried out through intracellular Ca^2+^ flux and results in changes in the level of expression of surface glycoproteins (including integrins) which can then act as receptors for platelet agonists and for adhesion proteins involved in platelet aggregation. After platelet activation, P-selectin translocates from α-granule membranes to the plasma membrane, and the GPIIb-IIIa complex on the plasma membrane undergoes a conformational change that exposes a fibrinogen binding site. Platelets then secrete the content of their granules (including Ca^2+^, Zn^2+^, coagulation factors and growth factors), adhere to subendothelial surface (GPIb-IX-V binds to vWF, GPIIb-IIIa binds to vWf or fibrinogen, and fibrin and other coagulation factors interact with the platelet surface), and aggregate to form a thrombus. Regulation of platelet function occurs through the action of the anti-aggregants prostacyclin and of nitric oxide, both of which are secreted by intact endothelial cells. Insulin inhibits platelet responses to stimuli through the P2Y_12_ pathway and sensitises platelets to the anti-aggregant effects of nitric oxide and prostacyclin.

The hyperglycaemia found in T1DM and T2DM results in: (1) reduced nitric oxide and prostacyclin production from the endothelium and nitric oxide production by platelets leading to an imbalance in the anti-aggregation mechanism [159]; (2) reduced insulin sensitivity of platelets in T2DM (or reduced insulin levels in T1DM), which leads to reduced inhibition of the P2Y_12_ pathway, itself resulting in a reduced platelet response threshold to stimuli and so an increased platelet reactivity [160,161]; (3) the glycation of proteins at the surface of platelets, leading to altered activity of, and signalling by, receptor proteins and to reduced platelet membrane fluidity, thus increasing platelet sensitivity to thrombin and platelet adhesion [7,159,162]; (4) increased activation of PKC which increases platelet activation [163]; (5) increased oxidative stress which activates the PKC pathway, but also leads to an increase in intracellular Ca^2+^ signalling and so to an increased platelet activation and aggregation [163,164]; (6) decreased production of antioxidants like glutathione, which has been linked to increased formation of thromboxane A2, leading to increased platelet activation [159,165]; (7) elevated basal Ca^2+^ levels in platelets and disturbed Ca^2+^ homeostasis which directly regulates platelet activation, platelet morphology, and initiation of coagulation [159,166]; (8) increased surface expression of glycoproteins such as GPIb and GPIIb/IIIa and increased activation of GPIIb/IIIa, leading to increased binding to vWF and fibrin(ogen), both resulting in increased platelet aggregation [159,167].

**Figure 4:**
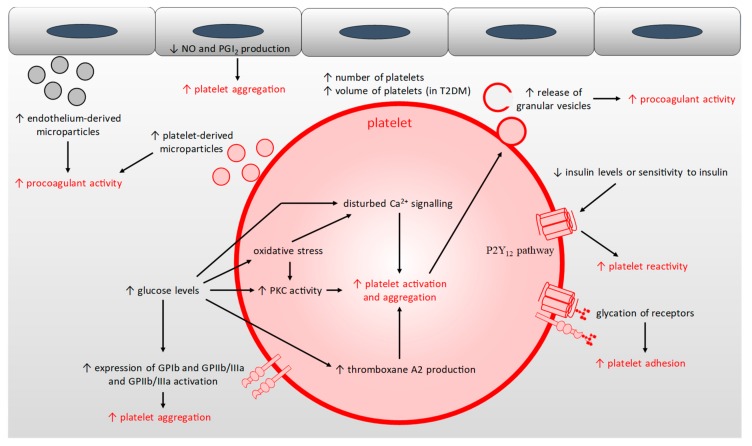
Mechanisms resulting in the hyper-reactivity, hyper-activation, aggregation and adhesion of platelets in T1DM and T2DM. Abbreviations used: NO, nitric oxide; PGI_2_, prostacyclin; PKC, protein kinase C; T2DM, type-2 diabetes mellitus.

Furthermore, the hyper-activation of platelets in individuals with diabetes means that they are consumed more rapidly such that platelet turnover is faster [159]. This leads to the generation of new platelets that are themselves inherently hyperactive [168]. In addition, in both T1DM and T2DM, platelet counts have been found to be higher [169]. This parameter responds positively to glycaemic control only in individuals with T1DM, while individuals with T2DM have a higher number of large platelets which display increased platelet reactivity [159,168,169]. Thus, in individuals with diabetes, platelets are more active, leading to increased adhesion, activation and aggregation and the increased production of platelet-derived microparticles [6,159]. Collectively, these changes result in an increased triggering of thrombus formation and an increased release of pro-coagulatory molecules by platelets such as Ca^2+^, Zn^2+^, fibrinogen, vasoconstrictors and oxidative reactive species which increase coagulation and the atherosclerotic process in both T1DM and T2DM (Figure 4) [159].

### 2.6. Pro-Coagulatory Microparticles

During cell growth, proliferation, activation and apoptosis, cells communicate through the release of extracellular vesicles. Microparticles are a heterogenous type of these vesicles with a diameter of 0–0.1 µm and whose content includes lipids, proteins and microRNAs depending on their origin [170]. The shedding of microparticles by cells is triggered notably by pro-inflammatory cytokines, AGEs, oxidative stress, LDLs and hyperglycaemia and their size, structure and content differ depending on the cell type and the stimuli triggering their formation [159]. Microparticles are constantly present in the blood, but many cardiovascular diseases are associated with elevated levels of these, especially microparticles derived from platelet and endothelial cells [171]. In T2DM, microparticle levels in the blood are increased; in particular, endothelial-derived microparticles enriched in CD31, CD62E, CD105 and CD106 [172], as well as platelet-derived microparticles enriched in fibrinogen (two-fold increase compared to non-diabetic individuals [173]), tissue factor (three-fold increase compared to non-diabetic individuals [173]) and P-selectin [174]. In addition, a proteomic analysis carried on microparticles taken from individuals with T2DM and controls has shown that in T2DM, proteins involved in platelet activation, cell adhesion, and inflammation are differentially expressed [175]. High levels of platelet-derived microparticles are associated with atherosclerotic progression and arterial thrombosis in individuals with T2DM [170]. Microparticle levels in T2DM are an independent predictor of adverse cardiovascular events (adjusting for age, gender, hyperlipidaemia, smoking and statin use) [170]. Individuals with T1DM also have elevated levels of endothelial- and platelet-derived microparticles and total levels of microparticles enriched with annexin V [176,177,178]. In T1DM, increased pro-coagulant activity was found to be associated with the total number of microparticles enriched with annexin V [177]. The levels of endothelial- and platelet-derived-microparticles as well as procoagulant activity directly correlated with HbA1c levels in individuals with T1DM [176,177,178].

Microparticles formed from platelets are extremely pro-thrombotic and facilitate thrombin generation [171]. These microparticles are enriched in tissue factor, constituting a “blood-borne” reservoir of this protein [171]. While tissue factor exposed at the blood vessel wall during coagulation initiates thrombus formation, the “blood-borne” tissue factor is involved in the propagation of coagulation [171]. Thus, the increase of “blood-borne” tissue factor found in T2DM contributes to the pro-thrombotic phenotype of the disease [170]. Such platelet-derived microparticles can be trapped in the developing thrombus through interactions of CD15, CD18 and tissue factor with the thrombus [170]. Furthermore, smooth muscle cell-derived highly pro-thrombotic microparticles enriched in tissue factor can be trapped inside atherosclerosis plaques and released during plaque erosion or rupture [179]. Microparticles then create a binding surface for further platelet recruitment and for fibrin after plaque disruption [170]. Microparticles derived from activated platelets can also activate other platelets by releasing arachidonic acid [180]. In addition to tissue factor, microparticles can induce thrombin generation in a factor XII-dependent manner (for erythrocyte- and platelet-derived microparticles) and in a factor XI-dependent manner (for erythrocyte-derived microparticles) [179,181]. Thus, the elevated levels of pro-coagulatory microparticles present in individuals with T1DM and T2DM participate in the high thrombotic risk associated with those diseases (Figure 4).

## 3. Differences in Thrombotic Risks Between T1DM and T2DM

Despite the different aetiologies of the diseases, numerous similarities exist in the hyper-coagulatory state found in individuals with T1DM and T2DM, as well as differences. Both diseases are characterised by hyperglycaemia, altered insulin metabolism, dyslipidaemia, endothelial dysfunction, oxidative stress and inflammation. However, individuals with T2DM have elevated FFA levels that are not found in T1DM, which can impact on the regulation of many physiological processes, including alterations in fibrin clot parameters, endothelial dysfunction, atherosclerotic plaque formation and cardiac lipotoxicity. In addition, insulin levels are often elevated in T2DM and accompanied with insulin resistance, while they are lowered in T1DM (although insulin resistance can also appear in T1DM [182]). In T2DM, β-cells can become damaged, leading to reduced insulin production and increased platelet reactivity [3]. Furthermore, both diseases are associated with differentially altered plasma concentrations of metal ions, leading to dysregulation of coagulation via distinct mechanisms. Finally, PAI-1 levels are elevated in T2DM but reduced in T1DM, even though both these diabetes types are associated with prolonged fibrin clot lysis times. These distinctions are very important and need to be taken into account during treatment of each disease. The similarities and differences in how T1DM and T2DM impact on coagulation are summarised in Table 2.

Few studies have looked at whether subtypes of T1DM and T2DM have an influence on these pro-thrombotic factors. Obese individuals with T2DM can be expected to have elevated plasma FFA levels compared to non-obese individuals with T2DM and we have already discussed the pro-thrombotic effects of FFAs. In addition, obese individuals with T2DM have higher risks of thrombosis than non-obese individuals with T2DM as they have delayed fibrinolysis, higher plasma concentrations of vWF and fibrinogen and higher levels of factor VII and factor VIII activity [183]. Individuals with T2DM who also have a genetic predisposition to T2DM are likely to be associated with some additional pro-thrombotic factors compared to individuals with T2DM without genetic predisposition; indeed, individuals with some genetic predisposition to T2DM but who have not developed T2DM have an elevated risk of cardiovascular disease [184,185]. Among individuals with T2DM, those who have a genetic predisposition to T2DM are also at higher risk of developing cardiovascular diseases [186]. Those “at risk” genes include genes involved in lipid oxidation (e.g., paraoxonase), antioxidation (e.g., superoxide dismutase) and anti-inflammation (e.g., adiponectin) [187]. When comparing non-fulminant T1DM and fulminant T1DM (a subtype of idiopathic T1DM defined by a short time period between the advent of symptoms and the onset, resulting from the rapid and complete breakdown of pancreatic β cells) over five years, one study found no difference in the development of microangiopathy complications [188], while another study which followed a higher number of individuals found higher microangiopathy incidence in the fulminant T1DM group compared to autoimmune T1DM [189]. Individuals with idiopathic T1DM were also found to have higher body mass index and LDL levels, a higher visceral adiposity index (an indicator of low-grade inflammation and cardiovascular risk) higher levels of obesity (and so presumably higher levels of plasma FFA and of the dysregulations they cause) and hypercholesterolemia and lower HDL levels compared to individuals with autoimmune T1DM [190]. The levels of micro and macrovascular complications were the same between the two groups [190]. Thus, more studies are needed to understand how the different subtypes of T1DM and T2DM differ in terms of cardiovascular biomarkers and thrombosis risk.

## 4. Current Treatment and Future Perspectives

Due to differences in how T1DM and T2DM impact upon coagulatory pathways, individuals with these diseases respond differently to drugs designed to reduce thrombotic risk and so special care needs to be taken in identifying the most suitable treatment regimen for an individual. The effects of anti-platelet drugs, lipid-lowering drugs and hypoglycaemic drugs on the coagulation system of individuals with diabetes has been reviewed in more depth by Alzahrani and Ajjan [4]. The current treatment for diabetes focuses on lifestyle changes to control glycaemia [191,192,193]. As shown in this review, hyperglycaemia is the origin of many changes in coagulation and good glycemia control has been shown to greatly reduce many of the symptoms, including normalising the plasma concentration of many coagulatory proteins, reducing platelet hyper-activation and aggregation, endothelial dysfunction and plasma levels of microparticles. If lifestyle changes are not sufficient, drugs can be prescribed. Many drugs help control blood glucose levels such as metformin or glipizide (a sulfonylureas type of glucose-lowering drug), which have been shown to reduce plasma levels of PAI-1 in monotherapy and to a greater degree when used in combination [194]. Lowering blood glucose levels reduces the risk of developing cardiovascular diseases in diabetes, but only up to a certain level, as hypoglycaemia has been shown to be pro-thrombotic too [63]. On its own, metformin has beneficial effects including weight loss (and so presumably a reduction in plasma FFA levels), improvements in haemostatic function (more efficient fibrinolysis and reduced clot formation tendency), reduced inflammation and oxidative stress, improved endothelial function and reduced atherosclerotic plaque formation. The latter includes inhibition of the conversion of monocytes to macrophages, reduced invasion of the arterial wall by inflammatory cells and reduced lipid uptake by activated macrophages within the atherosclerotic plaque [195]. Whether metformin influences cardiovascular disease risk is unclear. Two meta-analyses have shown that when metformin is used alone, it does not have a significant effect on cardiovascular disease risk in T2DM; however, these analyses mostly included short-term studies [196,197], while another meta-analysis and a study that followed patients over a longer period (the UK Prospective Diabetes Study) have shown beneficial effects [195,198,199]. Other classes of glucose-lowering drugs, such as sulfonylureas and dipeptidyl peptidase-4 (DPP-4) inhibitors, have also been found to have no beneficial effect on cardiovascular risk in T2DM [200,201,202]. DPP-4 inhibitors have been shown to inhibit platelet aggregation by interfering with tyrosine phosphorylation of the platelet plasma membrane Ca^2+^-ATPase channel, thus limiting the accumulation of intracellular Ca^2+^ [203]. They also improve endothelial nitric oxide signalling in the vasculature, thereby reducing endothelial dysfunction, and also reduce inflammation, atherosclerotic plaque formation and oxidative stress [204,205]. Another class of glucose-lowering drug, thiazolidinediones, has been shown to have some beneficial effects on cardiovascular disease risk but also increases the risks of congestive heart failure [200]. Recently, two new classes of glucose-lowering drugs, sodium–glucose cotransporter 2 (SGLT2) inhibitors and glucagon-like peptide-1 (GLP-1) receptor agonists, have been shown to have beneficial effects on cardiovascular disease risk in individuals with T2DM and established cardiovascular disease [200,201]. GLP-1 receptor agonists inhibit platelet aggregation and thrombus formation through enhanced nitric oxide production by activating endothelial nitric oxide synthase, thereby also reducing endothelial dysfunction. [206,207]. They also lower postprandial dyslipidaemia [207]. SGLT2 inhibitors are mostly beneficial in decreasing heart failure as they enhance cardiac cell metabolism, reduce cardiac fibrosis, inhibit Na^+^/H^+^ exchange in myocardial cells, modulate adipokine and cytokine production, improve ventricular loading conditions and decrease blood pressure [208,209].

Lipid-lowering drugs such as statins are also useful and used mainly to lower total cholesterol and LDL levels. In addition to their effects on LDL, lipid-lowering drugs used in the treatment of diabetes can have additional beneficial effects. Indeed, many of these drugs, including statins and fenofibrate, also affect FFA levels as we have reviewed before [5]. This is an important effect as FFAs regulate many physiological processes, including endothelial function. Furthermore, lipid lowering treatment with statins can limit the level of circulating microparticles by reducing thrombin generation and the expression of tissue factor, GPIIIa and P-selectin on platelet-derived microparticles in patients with peripheral vascular diseases [170,210]. They also increase the levels and anticoagulant activity of protein C, protein S and antithrombin, reduce the levels of pro-coagulatory proteins prothrombin, factor V, factor VII, factor VIII, factor IX and factor X, reduce the levels of anti-fibrinolytic PAI-1 and reduce the expression of tissue factor by endothelial cells [4,47,211]. However, statins have been reported to both increase [47] and decrease [211] the levels of antithrombin. Statins have been shown to reduce endogenous thrombin potential and thrombosis risk in T2DM and they reduce atherosclerotic risk through a reduction in inflammation and endothelial dysfunction [4]. Statins taken in combination with other LDL-lowering drugs such as ezetimibe or proprotein convertase subtilisin/kexin type 9 (PCSK9) inhibitors showed beneficial effects on cardiovascular disease risk [212]. In addition, drugs designed to increase HDL-cholesterol levels such as niacin, fenofibrate and CETP inhibitors have been designed. It is important to highlight though that these drugs fail to further decrease cardiovascular risk when administered to individuals already taking statins [137]. Nevertheless, fenofibrates also have some effect on the coagulation system, including decreasing levels of fibrinogen, tissue factor, factor VII and PAI-1 [4].

If therapeutics targeting glycaemia and lipid levels are insufficient at reducing cardiovascular risk markers in diabetes, anti-platelet drugs can also be prescribed [191,192,193]. It is well known that individuals with diabetes are less responsive to anti-platelet drugs, but nevertheless dual treatment with aspirin and the P_2_Y_12_ inhibitor clopidogrel is still advised [9]. Other strategies are being examined to lower thrombotic risk in individuals with diabetes, such as directly targeting the hypo-fibrinolysis found in diabetes, as reviewed by Kearney et al. [63]. These new drugs would inhibit thrombin activatable fibrinolysis inhibitor and PAI-1 and decrease the incorporation of α2-antiplasmin and C3 protein into fibrin clots.

As reviewed here, diabetes is a complex disease that strongly impacts on haemostasis and the risk of developing cardiovascular disease in multiple ways. These include alterations in the plasma levels of coagulatory proteins, metal ions and pro-coagulatory particles, lipid metabolism and composition, endothelial function and platelet reactivity. It is important to understand these mechanisms and how they differ between T1DM and T2DM in order to appropriately treat these diseases and to reduce thrombotic risk in affected individuals.

## Figures and Tables

**Table 1:** Summary of proteins that have exhibited an altered concentration or activity in individuals with type-1 diabetes mellitus (T1DM) or T2DM. Abbreviations used: PAI-1, plasminogen activator inhibitor-1; vWF, von Willebrand factor.

	T1DM	T2DM
Pro-coagulant proteins	↑ vWF [12]	↑ vWF [10,11,12]
	↑ prekallikrein [13]	↑ kininogen [23]
	↑ tissue factor procoagulant activity [17]	↑ kallikrein [14]
	↑ factor V [15]	↑ soluble tissue factor [16,24]
	↑ (activated) factor VII [15,17]	↑ factor V [15]
	↑ factor VIII [15]	↑ (activated) factor VII [15,16]
	↑ factor X [15]	↑ factor VIII [15,18]
	↑ factor XI [14]	↑ factor IX [18]
	↓ activated factor XII [27]	↑ factor X [15]
	↑ prothrombin [15]	↑ factor XI [14]
	↓ fibrinogen [22], ↑ fibrinogen in diabetic complications [21]	↑ (activated) factor XII [18,25]
		↑ factor XIII [26]
		↑ prothrombin [15]
		↑ fibrinogen [19,20]
Anticoagulant proteins	↓ antithrombin activity [49,50]	↑ antithrombin [18,33], ↓ antithrombin [32], ↓ antithrombin activity with bad glycaemic control [47]
	↓ protein C [15,19,28]	↓ protein C [15,19,29], ↓ protein C activity with bad glycaemic control [47]
	↓ protein S [30]	↓ protein S [30], ↓ protein S activity with bad glycaemic control [47]
	↑ tissue factor pathway inhibitor activity [49]	↑ tissue factor pathway inhibitor [16]
	↑ thrombomodulin [31]	↑ thrombomodulin [29]
Pro-fibrinolytic proteins	↑ tissue plasminogen activator in diabetic complications [37]	↑ tissue plasminogen activator [24]
	↓ plasmin activity [51]	
Anti-fibrinolytic proteins	↓ PAI-1 [39], ↓ PAI-1 activity [22,52]	↑ PAI-1 [10,19,20,24,38]
	↑ α2-antiplasmin [19,43], ↓ α2-antiplasmin [22]	↑ α2-antiplasmin [19]
	↑ thrombin-activatable fibrinolysis inhibitor [41]	↑ thrombin-activatable fibrinolysis inhibitor [40]
	↑ α2-maroglobulin [42]	↑ α2-maroglobulin [32,42]

↑ indicates an increase in concentration or activity, while ↓ indicates a decrease.

**Table 2:** Summary of the similarities and differences in how T1DM and T2DM impact on coagulation. Abbreviations used: AGE, Advanced glycation end-products; FFA, free fatty acid; HDL, high-density lipoprotein; LDL, low-density lipoprotein; PAI-1, plasminogen activator inhibitor; T1DM, type-1 diabetes mellitus; T2DM, type-2 diabetes mellitus.

	In T1DM	In T2DM	In both T1DM and T2DM
Coagulation	Reduced PAI-1 levels	Increased levels of anti-fibrinolysis proteins, including PAI-1	Increased levels of pro-coagulatory proteins
			Reduced anticoagulant activity
			Denser fibrin fibres, less porous fibrin clot, fibrin clot more resistant to fibrinolysis
Metal ions	Dysregulation of coagulation by Mg^2+^ deficiency	Possible dysregulation of coagulation by elevated Ca^2+^ levels	Possible dysregulation of coagulation by elevated ceruloplasmin levels
	Possible dysregulation of coagulation by Fe^3+^ supplements	Dysregulation of coagulation by altered zinc speciation	
		Possible dysregulation of coagulation by elevated iron levels	
Lipids	Unchanged or reduced HDL levels	Reduced HDL levels	Elevated levels of small dense LDL that favoured atherosclerotic plaque formation and endothelial dysfunction
	Reduced plasma FFA levels	Elevated plasma FFA levels causing the destabilisation of fibrin clot, metabolism dysregulation, atherosclerotic plaques and cardiac lipotoxicity	HDL dysfunction causing reduced HDL efflux, reduced anti-inflammatory effects and endothelial dysfunction
			Hypertriglyceridemia causing HDL and endothelial dysfunction
Endothelial dysfunction		Excess FFA levels causing endothelial dysfunction	Endothelial dysfunction causes reduced nitric oxide production, dysregulation of vasodilators and vasoconstrictors
			Formation of AGEs dysregulating nitric oxide synthase and protein synthesis by the endothelium, causing endothelial dysfunction
			Matrix metalloproteinases upregulation, causing inflammation, endothelial dysfunction, vascular remodelling, thrombus formation and atherosclerotic plaque formation and destabilisation
Platelets	Platelets have unchanged volume	Larger platelets	Higher platelet count
			Hyper-activation, adherence and aggregation of platelets
Microparticles	Elevated levels of endothelial- and platelet-derived microparticles correlated with HbA1c and associated with pro-coagulatory activity	Elevated levels of endothelial- and platelet-derived microparticles enriched in coagulation proteins and associated with atherosclerosis and thrombosis	

## Abbreviations

AGE	Advanced glycation end-products
CETP	Cholesterol ester transfer protein
DPP-4	Dipeptidyl peptidase-4
GLP-1	Glucagon-like peptide-1
HDL	High-density lipoprotein
LDL	Low-density lipoprotein
PAI-1	Plasminogen activator inhibitor-1
PKC	Protein kinase C
PCSK9	Proprotein convertase subtilisin/kexin type 9
RAGE	Advanced glycation end-product receptors
S1P	HDL-associated sphingosine-1-phosphate
SGLT2	Sodium–glucose cotransporter 2
T1DM	Type-1 diabetes mellitus
T2DM	Type-2 diabetes mellitus
vWF	von Willebrand factor
